# The Characteristics, Uses, and Biases of Studies Related to Malignancies Using Google Trends: Systematic Review

**DOI:** 10.2196/47582

**Published:** 2023-08-04

**Authors:** Mikołaj Kamiński, Jakub Czarny, Piotr Skrzypczak, Krzysztof Sienicki, Magdalena Roszak

**Affiliations:** 1 Department of Rheumatology District Hospital in Kościan Kościan Poland; 2 Department of the Treatment of Obesity, Metabolic Disorders, and of Clinical Dietetics, Poznań University of Medical Sciences Poznań Poland; 3 Faculty of Medicine, Poznan University of Medical Sciences Poznań Poland; 4 Department of Thoracic Surgery Poznan University of Medical Sciences Poznań Poland; 5 Department of Computer Science and Statistics Poznan University of Medical Sciences Poznań Poland

**Keywords:** Google Trends, oncology, malignancies, prophylaxis, celebrity, infodemiology, infoveillance, cancer, carcinoma, lymphoma, leukemia, multiple myeloma, sarcoma, internet, tumor, bias, quality

## Abstract

**Background:**

The internet is a primary source of health information for patients, supplementing physician care. Google Trends (GT), a popular tool, allows the exploration of public interest in health-related phenomena. Despite the growing volume of GT studies, none have focused explicitly on oncology, creating a need for a systematic review to bridge this gap.

**Objective:**

We aimed to systematically characterize studies related to oncology using GT to describe its utilities and biases.

**Methods:**

We included all studies that used GT to analyze Google searches related to malignancies. We excluded studies written in languages other than English. The search was performed using the PubMed engine on August 1, 2022. We used the following search input: “Google trends” AND (“oncology” OR “cancer” or “malignancy” OR “tumor” OR “lymphoma” OR “multiple myeloma” OR “leukemia”). We analyzed sources of bias that included using search terms instead of topics, lack of confrontation of GT statistics with real-world data, and absence of sensitivity analysis. We performed descriptive statistics.

**Results:**

A total of 85 articles were included. The first study using GT for oncology research was published in 2013, and since then, the number of publications has increased annually. The studies were categorized as follows: 22% (19/85) were related to prophylaxis, 20% (17/85) pertained to awareness events, 11% (9/85) were celebrity-related, 13% (11/85) were related to COVID-19, and 47% (40/85) fell into other categories. The most frequently analyzed cancers were breast (n=28), prostate (n=26), lung (n=18), and colorectal cancers (n=18). We discovered that of the 85 studies, 17 (20%) acknowledged using GT topics instead of search terms, 79 (93%) disclosed all search input details necessary for replicating their results, and 34 (40%) compared GT statistics with real-world data. The most prevalent methods for analyzing the GT data were correlation analysis (55/85, 65%) and peak analysis (43/85, 51%). The authors of only 11% (9/85) of the studies performed a sensitivity analysis.

**Conclusions:**

The number of studies related to oncology using GT data has increased annually. The studies included in this systematic review demonstrate a variety of concerning topics, search strategies, and statistical methodologies. The most frequently analyzed cancers were breast, prostate, lung, colorectal, skin, and cervical cancers, potentially reflecting their prevalence in the population or public interest. Although most researchers provided reproducible search inputs, only one-fifth used GT topics instead of search terms, and many studies lacked a sensitivity analysis. Scientists using GT for medical research should ensure the quality of studies by providing a transparent search strategy to reproduce results, preferring to use topics over search terms, and performing robust statistical calculations coupled with sensitivity analysis.

## Introduction

### Background

Globally, malignancies remain a significant contributor to morbidity and mortality [1]. Contemporary care of neoplastic conditions is a complex process, demanding precise molecular and radiological diagnostics, advanced treatment strategies, as well as psychological and social support [2]. Furthermore, we are progressively transitioning toward personalized medicine, in which increasingly more aspects of malignancy treatment are tailored to the patient [3-5]. This intricacy of care, coupled with the use of specialized vocabulary, can make patients with neoplastic conditions and their families feel overwhelmed [6,7], thus prompting them to seek additional information about their disease from readily accessible sources.

The internet has become a significant source of health-related information [8-10]. Research indicates that approximately 80% of individuals with cancer seek medical or health-related information on the web [11]. Many patients perceive this information available on the web as a beneficial complement to the care provided by their physicians [8]. In addition, access to the internet enables the process of locating specialized medical centers and physicians who can provide tailored care for specific malignancies [12].

Infodemiology, the science of the distribution and determinants of information in electronic media, has emerged as a critical field in health informatics [13-16]. It focuses on analyzing data from internet sources, mainly social media and search queries, to inform public health and policy. Infoveillance is a subset of infodemiology that involves the systematic monitoring of web-based information for public health purposes [13]. Using data from search queries and social media platforms, infoveillance provides real-time insights into health-related behaviors and trends, aiding in disease surveillance and public health policy development [17,18].

### Objectives

In their quest for relevant information, most internet users turn to search engines. In the Western world, Google holds a dominant position as the preferred search engine [19,20]. Google Trends (GT), a free and accessible tool, presents search statistics derived from Google’s search engine [21]. These statistics are quantified as relative search volume (RSV), which varies between 0 and 100. On this scale, 100 signifies the peak of interest (100% of the interest in a specific inquiry during a particular period and location), whereas 0 denotes an absence of interest (0%). GT, archiving data from January 2004, enables a concurrent comparison of up to 5 terms. Users can set a region either in a specific country or globally. GT distinguishes between search terms and topics in the search input. Search terms refer to the exact strings of text inputted in GT. GT can correspond the search term with a suggested topic, which includes all searches linked to that specific topic, regardless of the language used [22]. For instance, inputting the search term “cervix” will yield 3 matched topics: “Cervix,” “Neck,” and “Cervical Cancer.”

GT has emerged as a practical tool for researchers, enabling them to analyze Google users’ interests in specific search terms associated with various phenomena. Some researchers have discovered an association between Google searches for specific cancers and their incidence [23]. GT has also been used to gauge the effects of celebrity deaths owing to cancer on the public’s interest in that specific malignancy [24,25]. Other notable examples of such trends include the interest in melanoma in relation to its prevalence in the population [26] and the awareness of the importance of colonoscopy in particular countries [27]. Most recently, Google query statistics have been leveraged to evaluate the decline in interest in cancer screening during the COVID-19 pandemic [28-30].

As the volume of GT studies has increased rapidly, it has become challenging to review all studies comprehensively at once [31]. However, a review can be more focused and examine GT studies within a specific health domain. Moreover, new types of studies have emerged, such as analyses of interest in health-related queries during a pandemic and investigations into the effects of educational campaigns or the death of a celebrity owing to cancer on public awareness in specific countries. Conducting a systematic review of GT studies in oncology is important because it can reveal insights into how digital search behaviors reflect public interest and concerns regarding cancer. In turn, this can guide public health messaging, early detection efforts, and resource allocation. Furthermore, a review in this area could bridge the gap between digital epidemiology and traditional oncology research, providing a more nuanced understanding of public engagement with cancer-related information. To date, a few reviews have characterized medical research using GT [31-33]. However, none of them explicitly focused on oncology. A systematic review may elucidate the use of GT in cancer-related studies, highlight its limitations, and establish best practices for future research.

We aimed to systematically characterize studies related to oncology using GT to describe its utilities and biases.

## Methods

### Overview

This was a systematic review. We followed the PRISMA (Preferred Reporting Items for Systematic Reviews and Meta-Analyses) 2020 guidelines on systematic review [34]. The PRISMA 2020 checklist is presented in Multimedia Appendix 1 [34].

### Study Selection

We included all studies using GT to analyze queries related to malignancies, solid mass tumors, blood cancers, or their screening methods (Textbox 1). We excluded studies using only other Google tools, for example, Google AdWords, and those written in non-English languages. Moreover, we excluded conference abstracts from consideration. The abbreviated descriptions often provided in these abstracts do not permit the reconstruction of the necessary search inputs that are crucial for evaluating the methodology of a study using GT.

**Criteria for inclusion of studies using Google Trends in the review.**

**Inclusion criteria**

- Used tool: Google Trends
- Health domain: solid tumor, lymphoma, leukemia, multiple myeloma, and their screening method
- Language: English
- Type of paper: original research, brief report, letter to the editor, etc

**Exclusion criteria**

- Used tool: other tools related to search engines statistics than Google Trends
- Health domain: not using at least one search term or topic related to malignancy or their screening method
- Language: non-English
- Type of paper: conference abstract

### Search Strategy

**Figure 1:**
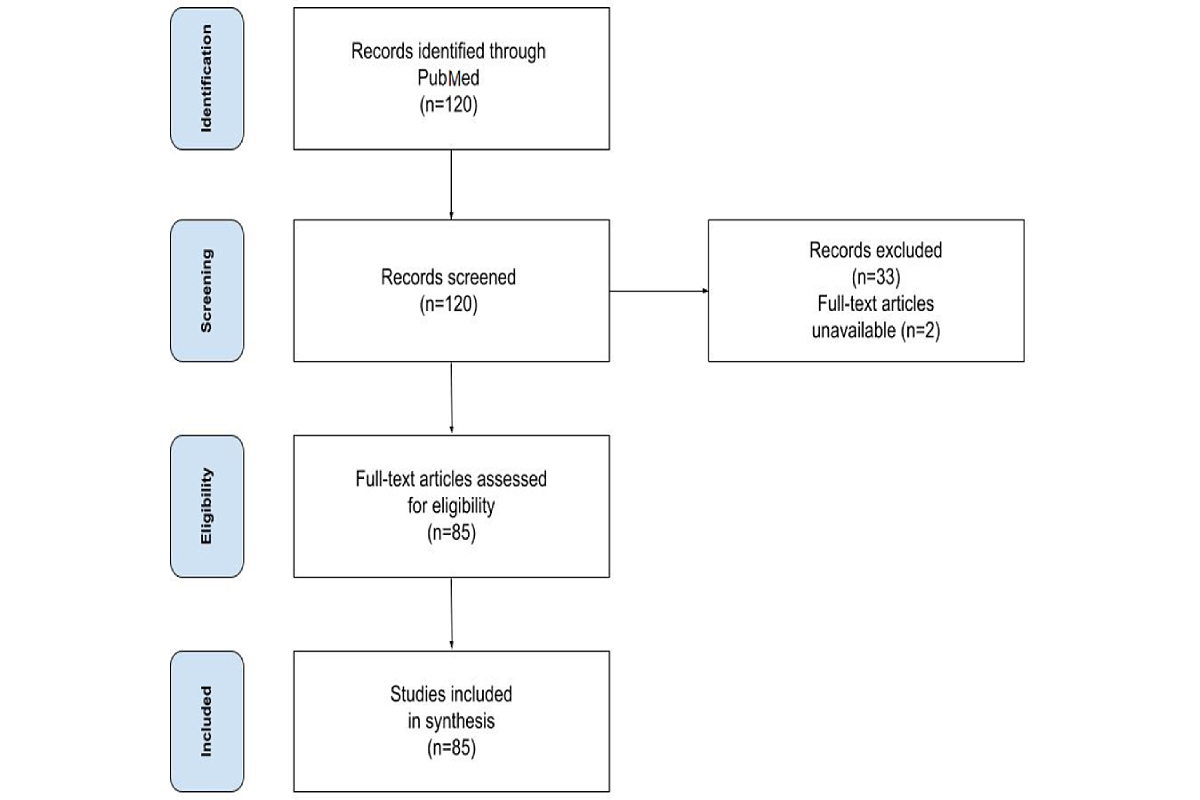
PRISMA (Preferred Reporting Items for Systematic Reviews and Meta-Analyses) flow diagram.

We used the PubMed search engine to search for studies on oncology-related GT analysis. The search was performed on the PubMed engine on August 1, 2022. The PubMed search input was the following: “Google trends” AND (“oncology” OR “cancer” OR “malignancy” OR “tumor” OR “lymphoma” OR “multiple myeloma” OR “leukemia”). Two authors (JC and MK) independently screened the titles and abstracts of potential articles. All discrepancies were referred by PS. The PRISMA flow diagram is presented in Figure 1. We obtained 120 studies for the title and abstract screening. Furthermore, we excluded 33 records, 2 were additionally excluded because of inaccessible full-version text, and 85 articles were thoroughly read. Finally, we included 85 studies for the final review.

### Evaluation of Studies

The included articles were carefully read and analyzed by one of the authors (JC) who retrieved all essential details. We collected the following details: title, DOI code, authors’ countries, year of publication, aims, aims classification, study category (prophylaxis, awareness month, celebrity death, COVID-19, and others), malignancies considered, analysis of topics or search terms, regions and period analyzed, statistical reproducibility method used, confrontation of GT results with real-world data (eg, with epidemiological data), principal findings, and sensitivity analysis. We classified aims (causal interference, description, surveillance, and other) based on the classification used in the review by Nuti et al [33]. After the preliminary screening of the included articles, 2 authors (MK and JC) formulated a classification scheme for the studies. The main goal of the classification was to distinguish the particular group of studies that used GT. This scheme, which was later approved by author PS, divided all the studies into 5 distinct categories: those related to prophylaxis methods; those related to awareness events (such as Pink October); those related to a celebrity (eg, a death or announcement of cancer diagnosis); those related to the COVID-19 pandemic; and finally, a category for nonspecific studies, which were classified as “Others.” The reproducibility of the study was determined by the authors’ transparency in providing specific details about their search input. This includes information if the search terms or topics were used, the region analyzed, the period of analysis, and whether countries with low or no low search volume were included if the region was set to “Worldwide.” These details are crucial for reproducing the study’s results. The complete characteristics of the studies are presented in Multimedia Appendix 2.

Studies using GT are retrospective and involve aggregated data of Google users without details, for example, sex and age. Nuti et al [33] addressed 2 sources of potential bias in GT studies: search strategy and validation of the studies. In this study, we analyzed the following sources of bias: (1) using search terms instead of topics, (2) lack of confrontation of GT results with real-world data, and (3) absence of a sensitivity analysis.

We conducted a descriptive statistical analysis. All categorical variables were presented as numbers and percentages, whereas all numerical variables were expressed as medians along with their IQRs. Data manipulation, calculations, and visualizations were performed using the R-programing language 3.6.1 (R Foundation for Statistical Computing).

### Ethical Considerations

This is a systematic review; thus, the paper did not require ethical committee’s approval.

## Results

### Countries

We included 85 articles for our final analysis. Furthermore, 10 examples of the included studies are presented in Table 1. Of the 85 articles, in 47 (55%) articles, at least 1 author was from the United States. Furthermore, at least 1 author in 5 articles was affiliated with institutions in Australia, Brazil, or the Philippines. Furthermore, 4 articles each had at least 1 author from Germany, Ireland, Turkey, or the United Kingdom. Three articles included authors from Austria, China, Italy, or Japan. In 2 articles, at least 1 author was from Canada, France, India, or Malaysia. Finally, each article had an author from Iran, Kuwait, the Netherlands, New Zealand, Poland, Portugal, Romania, or Spain. None of the authors were from Africa or Central America.

**Table 1:** Examples of studies related to malignancies using Google Trends.

Study, year	Aims	Aims classification	Study category	Search inputs	Region analyzed	Period analyzed	Statistical analysis	Confrontation with real- world data	Principal findings
Mondia et al [35], 2022	Description of the pattern of web search queries of the keywords related to neoplasms of the CNS^a^	Description	Celebrity-related	Search terms: “Brain tumor” (also as disease), “Brain cancer,” “Central nervous system (CNS) tumor,” “glioma” (also as topic), “Glioblastoma” (also as genetic disorder), “astrocytoma” (also as topic), “oligodendroglioma,” “CNS Lymphoma,” “Medulloblastoma” (also as topic), “meningioma” (also as medical condition), “temozolomide” (also as medication), and “oligodendroglioma” (also as topic)	Worldwide	January 2004 to January 2021	Peaks analysis	Yes	“Brain tumor,” “brain cancer,” “glioblastoma” and “glioma” had the highest search volume. RSV^b^ from Google Trends. There were no observable trends that could correlate to the rising numbers of brain tumor cases worldwide with the global interest in brain tumors.
Greiner et al [36], 2021	Assessment of how COVID-19 affected public interest in mammography, colonoscopy, and HPV^c^. Assessment of the efficacy of using public interest in cancer screenings	Description	COVID-19	Search terms: “colonoscopy,” “mammogram,” “HPV,” and “pap smear”	Worldwide	September 6, 2015, to August 30, 2020	Peaks analysis, forecasting	No	Public interest in cancer screenings decreased precipitously at the onset of the COVID-19 pandemic, but the decrease in interest in breast and colon cancer screenings slightly underestimated the actual screening use numbers. Google Trends estimated the decrease in mammogram use as 25.8% below the actual value. Similarly, Google Trends estimated the decrease in colon cancer screening use as 9.7% below the true value.
Naik et al [37], 2021	Quantification of the impact of Chadwick Boseman’s death on web-based search interest in colon cancer, checking whether there was an increase in interest in regions of the United States with a greater proportion of Black American residents	Causal interference, description	Celebrity-related	Topics: colorectal cancer, colon cancer screening; terms: colonoscopy, stool test, diagnosis, stool, symptoms, signs, anemia, risk, men, age, black (-panther), African American, treatment, survival, and death	United States	September 2, 2018, to November 29, 2020	Peaks analysis, correlation	Yes	The observed RSVs for the topics of colorectal cancer and colon cancer screening increased by 598% and 707%, respectively, and were on average 121% and 256% greater than expected during the first 3 months after Boseman’s death.
Pantel et al [38], 2021	Determination of the impact of National Colorectal Cancer Awareness Month on rates of screening endoscopies and public interest in colorectal cancer	Surveillance	Awareness month, prophylaxis	Search terms: “colorectal cancer,” “colorectal cancer symptoms,” and “colorectal cancer screening”	United States	January 2004 to July 2019	Correlation, others	Yes	Colorectal cancer screening endoscopy rates were not impacted by the specific month of the year and these rates had no seasonality. However, Google searches related to colorectal cancer were significantly impacted by month of the year, specifically March, with significant seasonality observed in the data. National Colorectal Cancer Awareness Month is associated with an increased public interest in colorectal cancer based on users’ Google search trends.
Faoury et al [39], 2019	Review of Google Trends as a method for investigating internet-based information-seeking behavior related to throat cancer in terms of quantity, content, and thematic analysis	Description	Others	Search terms: “throat cancer,” “cancer,” “HPV,” “laryngeal cancer,” and “head and neck cancer”	Worldwide	2004 to 2015	Peaks analysis	No	Three important peaks in searches for “throat cancer” (and “HPV”) were identified: the first and greatest increase in interest in September 2010, with peaks in June 2013 and in October 2011 also. When comparing “throat cancer” with “laryngeal cancer” and “head and neck cancer,” it was found that there was a significant correlation between the search terms “throat cancer” and “laryngeal cancer” in terms of peaks and timeline changes.
Phillips et al [23], 2018	Characteristics of the relationship between cancer incidence and Google search volumes in the United States for 6 common cancers. Evaluation of the association of search activity with cancer-related public events and celebrity news coverage	Surveillance	Celebrity-related, others	Search terms: “breast cancer,” “prostate cancer,” “colon cancer,” “lung cancer,” “uterine cancer,” and “leukemia”	United States	2004 to 2013	Correlation	Yes	The search volume measured over time noted the term “Dental caries” to be the most searched in Japan, “Gingivitis” in Jordan, “Oral Cancer” in Taiwan, “No Teeth” in Australia, “HIV symptoms” in Zimbabwe, “Broken Teeth” in the United Kingdom, “cleft palate” in the Philippines, and “Toothache” in Indonesia, and the comparison of top 5 searched terms provided the result “Gingivitis” with highest search volume.
Rosenkrantz et al [40], (2016)	Identification of geographic and temporal patterns related to the frequencies of web searches within the United States for information on imaging-based cancer screening tests	Causal interference	Prophylaxis	Search terms: “breast cancer,” “mammography,” “tomosynthesis,” “colon cancer,” “virtual colonoscopy,” “lung cancer,” “lung cancer screening,” “prostate cancer,” “prostate MRI,” and “prostate MRI biopsy”	United States	January 2004 to December 2014	Secular trend, peaks, correlation, others	No	Searches for “mammography” decreased slightly overall, although they peaked in October (Breast Cancer Awareness Month) in most years and spiked in November 2009. Instead, the frequency of searches for “lung cancer screening” decreased slightly from 2006 through 2010, increased rapidly from 2011 through 2014, and exhibited a spike in November 2010 (when the results of the National Lung Screening Trial were released).
Noar et al [25], 2013	Quantification of the effects of pancreatic cancer public figure announcements on web-based cancer information seeking and cancer media coverage	Causal interference	Celebrity-related	Search terms: “pancreatic cancer” or “pancreatic cancers” (pancreatic cancers) and “cancer” or “cancers”	United States	2006 to 2011	Peaks analysis, correlation	No	Most public figures’ pancreatic cancer announcements corresponded with no appreciable change in pancreatic cancer search queries or media coverage. In contrast, Patrick Swayze’s diagnosis was associated with a 285% increase in pancreatic cancer search queries, though it was only weakly associated with increases in pancreatic cancer media coverage. Steve Jobs’ death was associated with a 197% increase in pancreatic cancer queries and a 3517% increase in pancreatic cancer media coverage.

^a^CNS: central nervous system.

^b^RSV: relative search volume.

^c^HPV: human papillomavirus.

### Trends and Journals

The first study that used GT in cancer-related research was published in 2013. In the ensuing years, there has been a significant surge in the annual number of such publications. This trend culminated in 20 publications each in 2020 and 2021. Of the 85 articles, 23 (27%) studies were published in a journal dedicated to oncology, 9 (11%) in a journal about public health issues, 8 (9%) in a journal about dermatology, 18 (21%) in journals with a broad scope (eg, PloS One, BMJ Open, Cureus), and 28 (33%) in other types of journals. Furthermore, 38% (32/85) of the articles were published in open-access journals.

### Main Topics and Malignancies

**Figure 2:**
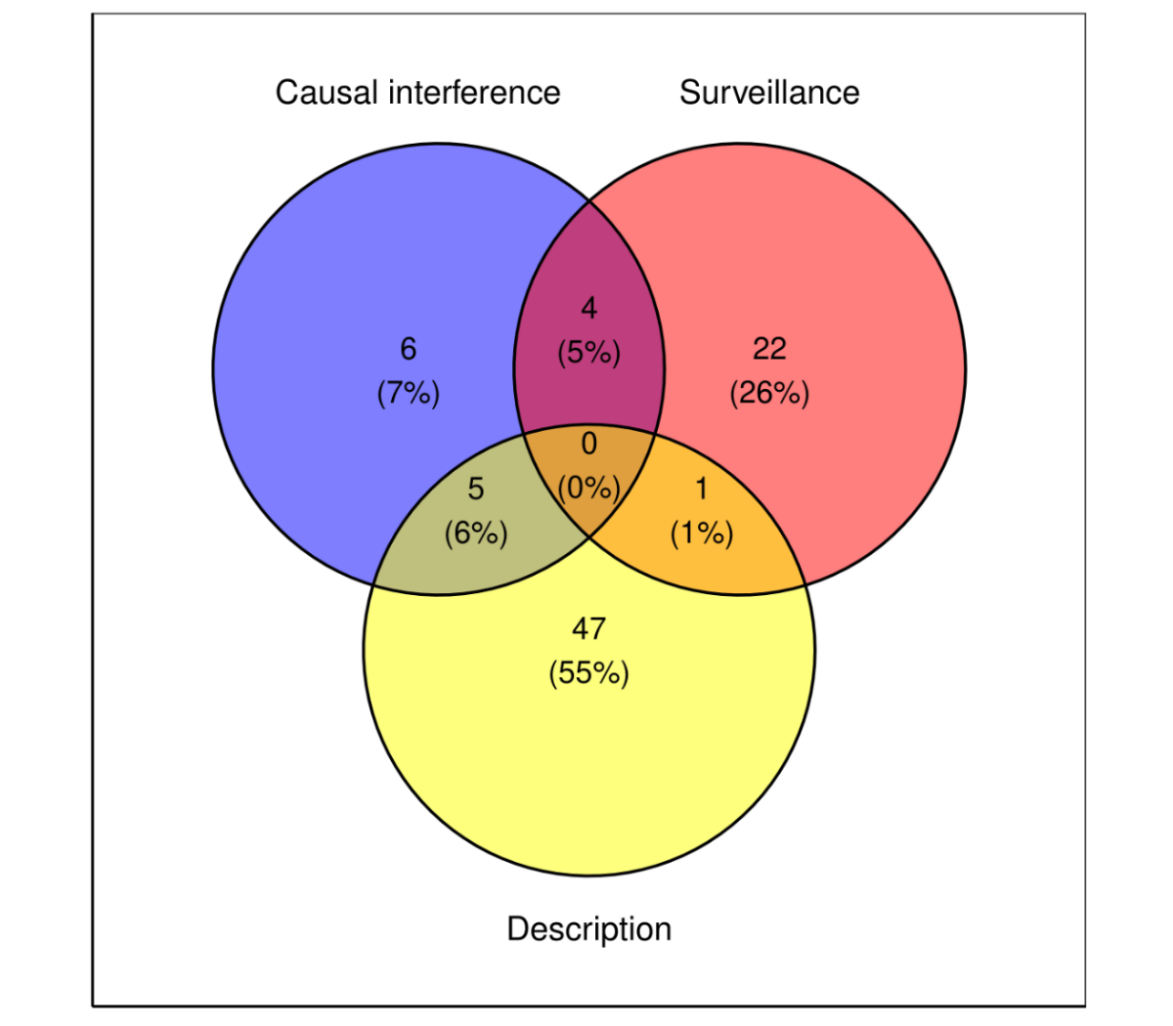
Classifications of the aims of the studies included in the review.

More than half (47/85, 85%) of the studies had descriptive characteristics, mainly characterizing temporal and regional trends (Figure 2). For example, Zhang et al [41] described the seasonal interest of Google users in “tobacco” and “lung cancer” in Australia, New Zealand, the United Kingdom, and the United States. Approximately 30% (27/85) of the papers aimed to conduct surveillance analysis. An example of a surveillance study is Brazilian research that evaluated the association between Google searches pertaining to breast cancer and mammograms and the actual number of diagnosed cases and mammograms conducted across various states in Brazil [32]. Finally, one of the 6 papers assessed the causal interference between specific events and Google searches. For instance, Noar et al [25] analyzed the effects of public figure announcements of diagnosis or death owing to pancreatic cancer on Google queries (Table 1).

**Figure 3:**
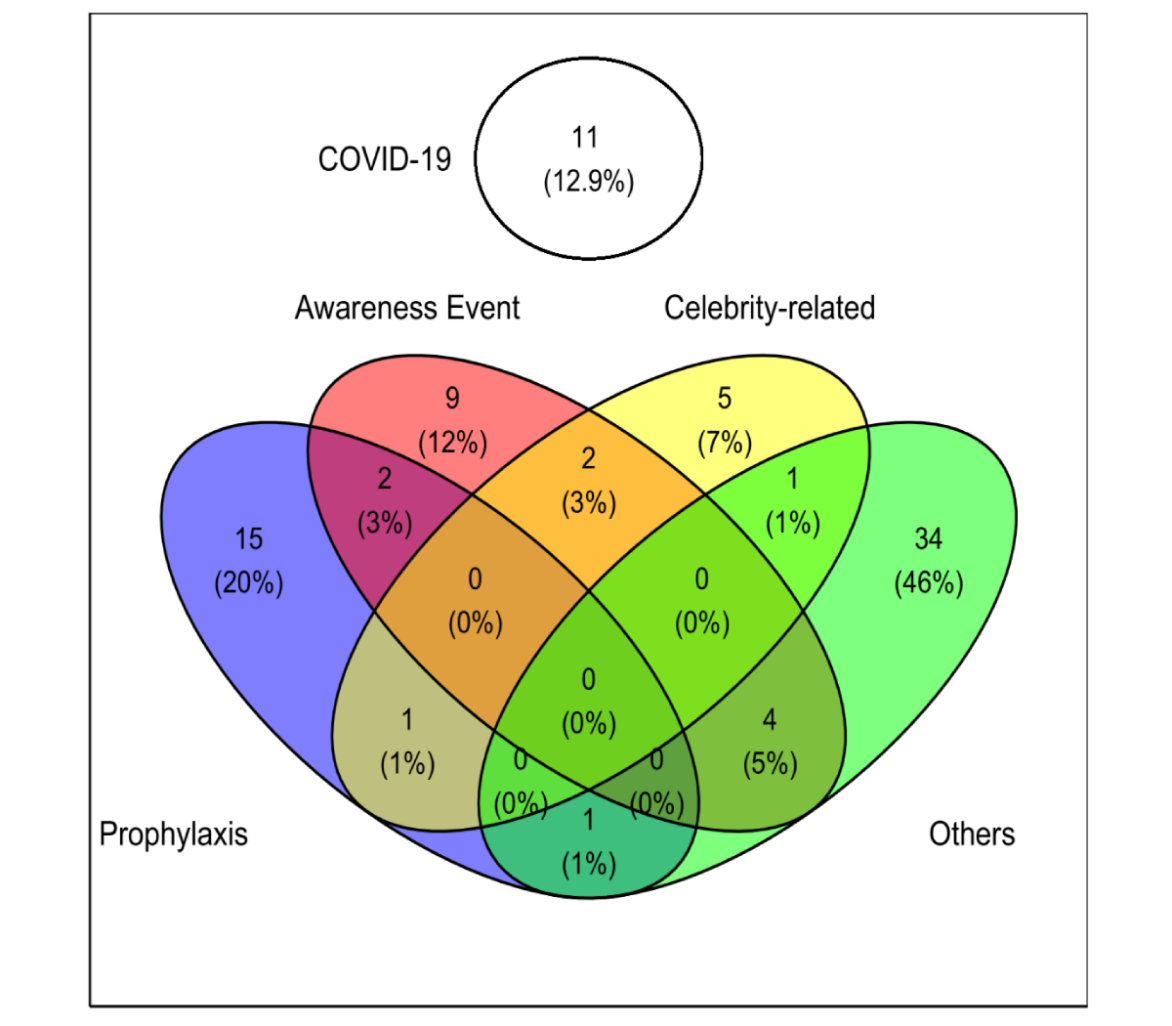
Categories of the studies included in the review.

We classified 22% (19/85) studies as related to prophylaxis, 20% (17/85) as awareness events, 11% (9/85) as celebrity-related, 13% (11/85) as related to COVID-19, and 47% (40/85) as others (Figure 3). An example of a study on prophylaxis was the research by Kaminski et al [27], who found an association between the burden of colorectal cancer and interest in colonoscopy among Google users in 60 countries. Several studies have analyzed the association between GT statistics and awareness month; for example, Pink October represents the breast cancer awareness month [36], and March is an awareness month for colorectal cancer in the United States [38]. Announcements of the death of celebrities owing to cancer may increase the number of Google queries on cancer, which was reported after the death of Chadwick Boseman owing to colorectal cancer (Table 1) [24,37]. In recent years, many researchers have analyzed the effects of the COVID-19 pandemic on the interest of Google users with many malignancies. For example, Adelhoefer et al [42] found that interest in many malignancies decreased during the pandemic’s first months [42].

The most frequently analyzed cancers were breast cancer (n=28), prostate cancer (n=26), lung cancer (n=18), colorectal cancers (n=18), skin cancers (n=16), and cervical cancer (n=14). In turn, the least frequently were mesothelioma (n=1), penile cancer (n=1), stomach cancer (n=2) or the biliary tract cancer (n=2).

### Statistical Analysis

We found that 20% (17/85) of the studies used GT topics instead of search terms. In total, 28% (24/85) of studies incorporated GT statistics for all available countries. In addition, 39% (33/85) of the studies were conducted exclusively for the United States, whereas 35% (30/85) of the studies focused on other countries or a combination of different countries, including the United States. A total of 47% (40/85) of the studies used the most prolonged period available in GT from January 1, 2004.

Most (79/85, 93%) of the studies provided all search input details to reproduce their results. Overall, 40% (34/40) studies compared the GT statistics with real-world data. Almost two-thirds (55/85, 65%) of the studies performed correlation analysis and more than half (43/85, 51%) performed peak analysis. The less popular statistical approaches included secular trends analysis (9/85, 11%), seasonal trends analysis (6/85, 7%), forecasting (4/85, 5%), and others (24/85, 28%).

The authors of only 11% (9/85) of the studies performed a sensitivity analysis. Most studies compared the RSV of analyzed terms to the medical [35,42-45] and nonmedical search terms [46]. Another convincing sensitivity analysis involved comparing the results between different countries [26] or between countries from the northern and southern hemispheres [47].

## Discussion

### Overview

We systemically reviewed all studies on malignancies using GT available in PubMed until August 1, 2022. GT appears to be a feasible tool for the infodemiology of cancers. Most of the analyzed studies could be reproduced but had many sources of bias.

### Principal Findings

Our study found that the number of studies using GT in oncology has constantly been increasing. Most of the included papers came from the Western world, and we did not find any papers from Africa or Central America. Moreover, many available studies have successfully used data from the Global South, for example, Africa [48,49]. Previous studies observed that many low-income countries have a low search volume for popular health topics, such as pain [50] or diets [51]. This could be related to the lower access to the internet in the Global South.

Furthermore, low-income countries have far fewer productive research facilities than the Western world [52]. However, the number of people using the internet is rapidly increasing in low-income countries [53]. Therefore, we may expect more data on Google searches in these regions to be available shortly.

The included articles were published in journals with various scopes. Moreover, approximately 1 in 3 papers were published in an open-access journal. It is tempting to hypothesize that articles using GT are usually viewed as a curios subject and often published in wide-scope journals.

In our systematic review, we used the classification of GT articles previously used by Nuti et al [33]. In this study, we observed that most oncological studies using GT were of a descriptive character, a total of <20% analyzed causal interference, and up to 30% focused on surveillance. Nuti et al [33] reported that 39% of the studies used GT for description, 34% for surveillance, and 27% for causal interference. The proportions of both reviews seem to be similar. However, the review by Nuti et al [33] included all GT studies on health phenomena from 2009 to 2013, whereas we applied narrower inclusion criteria but included all studies until mid-2022. Nuti et al [33] defined descriptive studies as those “aimed to describe temporal or geographic trends and general relationships, without reference to a hypothesized causal relationship.” Therefore, we propose that descriptive studies are generally easier for researchers to perform, which explains their popularity.

The analyzed studies differ in terms of study category, which presents many ideas for using GT in oncology-related studies.

A large group of papers took the form of prophylaxis studies. They mainly focused on assessing the interest in oncological screening and a wide range of preventive activities and risk factors for common neoplasms. Most studies analyzed the interest of Google users in cancer screening in the United States. These studies concerned, among others, topics related to the prevention of melanoma and various risk factors of skin cancer known from the literature [54-57]. Our systematic review also included 2 papers that analyzed the interest in cancer prevention in Malaysia regarding melanoma [56] and breast cancer [58]. Interest in cervical cancer has been examined in Portugal [59] and Ireland [60]. However, it should be emphasized that, apart from the study by Kaminski et al [27] on Google searches for trends and terms related to colonoscopy, no other study has extensively used these topics and analyzed screening programs in many countries.

An interesting application of GT is examining the relationship between a celebrity’s cancer diagnosis and global interest in that specific malignancy. Generally, the announcement of a celebrity’s cancer diagnosis, especially when coupled with premature death, significantly boosts public interest in that specific cancer type. For instance, increased interest in colorectal cancer and colon cancer screening has been linked to the death of actor Chadwick Boseman [37]. Similarly, the diagnoses and deaths of actor Patrick Swayze [25] and Apple cofounder Steve Jobs [25] because of pancreatic cancer, as well as actor Ben Stiller’s prostate cancer diagnosis [61], triggered heightened interest. Moreover, GT analysis is not limited to cancer-related celebrity deaths. Another example in the scientific literature is the study of public interest following the death of actor Harold Allen Ramis because of complications from autoimmune inflammatory vasculitis [62].

A different approach involves using GT to examine whether social campaigns or cancer awareness months are associated with the interest fluctuations of Google users in specific malignancies. First, GT is a widely available free tool for checking the effectiveness of health promotion events. Data from several studies have shown a correlation between the cancer awareness campaigns and the increased public interest in oncological screening. Such observations have been reported for Pink October and breast screening in Malaysia [58]. Furthermore, several studies have confirmed that events such as breast cancer awareness month were associated with increased interest in this topic among Google users [63]. Studies confirmed that compared with other months, there was an increase in searches for topics related to breast cancer and mammography in October in all countries [64,65].

However, similar campaigns for lung or prostate cancer did not show a similar relationship [63]. Patel et al [64] observed that cancer awareness campaigns aimed at men were not associated with a significant increase in interest in topics related to screening, risk factors, or cancer itself. Gender differences might contribute to these findings, as women, who are frequent internet users, are often portrayed as more likely to search for health topics and be more aware of internet use in this area [66]. Interestingly, studies analyzing data from all countries show the lack of intended effectiveness and increased interest in many cancers [67-69], and studies focusing on specific countries such as Brazil [70] and New Zealand [71] have reported increased interest in cancers, such as glaucoma, prostate, and lung cancers. Finally, not only have awareness events related to malignancies been investigated with GT, but a similar study on the effect of World Sepsis Day was conducted as well [72].

In the context of the COVID-19 pandemic, several studies have analyzed Google users’ interest in cancers, comparing the prepandemic period with the first months or years of the pandemic. These studies have generally found a decrease in interest in many cancers and their screening programs. This decrease in RSVs for cancer-related search terms paralleled a decrease in the number of diagnostic procedures and new cancer diagnoses [42,73-75]. These findings highlight how GT can be used to study emerging public health problems such as the COVID-19 pandemic. For instance, we present a series of GT studies that suggest that the COVID-19 pandemic might have reduced cancer awareness, potentially leading to increased mortality during the pandemic because of delayed diagnoses. Notably, some of these GT observations were corroborated by the real-world data.

Interestingly, the most commonly analyzed cancers are those (1) with screening programs or (2) the highest prevalence worldwide [40,76]. The search terms representing rare malignancies could be queried for by a small population of Google users. Therefore, the trends of rarely typed search terms can be susceptible to irregular fluctuations without specific secular or seasonal patterns [39]. The trend of search terms with a low search volume could be problematic for the statistical analysis and interpretation of the results. This problem can be mitigated by matching the search terms of a topic in the GT search engine to include more significant regions and queries related to the topic [22]. However, only one in 5 included GT studies admitted to using topics instead of search terms.

Correlation and peak analyses were the most prevalent among the included studies. These methods are widely used and easy for readers to understand. However, correlation analysis only allows for the detection of associations, and many outcomes may represent incidental findings if they are not compared with real-world data or are supported by sensitivity analysis. Regrettably, only a few studies have analyzed secular or seasonal patterns, and only 4 performed forecasting. Our findings are similar to those of Mavragani et al [31], who also found that correlation analysis was the most prevalent and forecasting analysis was the least prevalent among studies using GT for analyzing health phenomena [31].

Furthermore, 40% (34/85) of the papers compared their results with real-world data, and only 9 reviewed studies applied sensitivity analysis [26,42-47]. In our opinion, many GT research projects, if not compared with real-world data (eg, epidemiological data) or not supported by appropriate sensitivity analyses, serve merely as a form of curiosity. GT data cannot serve as a foundation for new recommendations or epidemiological or eHealth tools in their current form. In this form, the data from GT only incites curiosity, offering, at most, a new perspective on some epidemiological issues. However, GT may provide insights into health phenomena that would otherwise require large-scale observations with uncertain clinical significance.

Whether GT data will be helpful in oncology is, in our opinion, difficult. GT data seem to be much better at dealing with more straightforward issues, for example, analyzing queries representing specific symptoms. Another interesting approach is the analysis of the effects of specific events (COVID-19 or celebrity death) on the interest of Google users in health phenomena. The question remains: under what circumstances is GT data analysis more convenient than conducting an extensive survey? Future studies should focus on translating knowledge from GT data into practical implications for modern oncology.

### Strengths and Practical Implications

To the best of our knowledge, this is the first study to explore the use of GT specifically in oncology and hematology. We characterized various studies presenting readers’ feasibility of GT in research related to oncology and hematology. We identified methodological weaknesses in the analyzed reports. Papers using GT data should not only report search inputs appropriately but also report them as proposed by Nuti et al [33]. Furthermore, using topics allows the inclusion of more regions, which increases the value of the results. We also suggest that GT data should be either confronted with real-world data or, if seasonality is analyzed, searches in both hemispheres should be compared. Another interesting approach to sensitivity analysis in GT studies was presented by Gillis et al [77], who proposed benchmark search terms to exclude random seasonal patterns related to the academic year. Finally, it is worth mentioning that there are no strict rules for sensitivity analysis, but even reanalysis, which excludes certain periods or regions, can increase confidence in the obtained results.

### Limitations

The authors are aware of the limitations of this systematic review. Our systematic review incorporated retrospective studies across a wide range of topics, which presented challenges in their uniform classification. This limited our analysis to descriptive statistics. Furthermore, the field lacks established standards for conducting systematic reviews of studies that use GT data. The novelty of the tool necessitates that researchers exercise considerable diligence. However, this is one of the first systematic reviews of GT and methodological practices are still evolving. In our review, we did not analyze whether the exact results of the papers could be reproduced or whether the authors drew appropriate conclusions. Finally, we opted to include only those studies that were accessible via PubMed. We assumed that the inclusion of (1) conference abstracts and (2) studies from less recognized journals could potentially introduce a selection bias. The abbreviated format of conference abstracts may not provide the necessary details about search inputs, and studies from niche journals may lack rigorous methodology. Consequently, such an approach could lead to the underestimation of the quality of research using GT in relation to malignancies.

### Conclusions

The number of studies related to oncology using GT data increases from year to year. The studies included in this systematic review demonstrate a variety of topics, search strategies, and statistical methodologies. The most frequently analyzed cancers were breast, prostate, lung, colorectal, skin, and cervical cancers, potentially reflecting their prevalence in the population or public interest. Although most researchers provided reproducible search inputs, only one-fifth used GT topics instead of search terms, and many studies lacked a sensitivity analysis. Scientists using GT for medical research should ensure the quality of studies by (1) providing a transparent search strategy to reproduce results, (2) preferring to use topics over search terms, and (3) performing robust statistical calculations coupled with sensitivity analysis.

## Appendix

Multimedia Appendix 1 PRISMA (Preferred Reporting Items for Systematic Reviews and Meta-Analyses) 2020 checklist.

Multimedia Appendix 2 Full characteristics of the included studies.

## Abbreviations

GT: Google Trends
PRISMA: Preferred Reporting Items for Systematic Reviews and Meta-Analyses
RSV: relative search volume

## Data Availability

The data set is presented in Multimedia Appendix 2.
