# Supplementary material for: The Characteristics, Uses, and Biases of Studies Related to Malignancies Using Google Trends: Systematic Review
Source: J Med Internet Res. 2023 Aug 4;25:e47582. doi: 10.2196/47582 (PMC10439473; doi:10.2196/47582)
Supplement: Multimedia Appendix 1 [file jmir_v25i1e47582_app1.pdf]

Supplementary File 1

PRISMA 2020 Checklist

Section and Topic	Item #	Checklist item	Location where item is reported
**TITLE**			
Title	1	Identify the report as a systematic review.	The characteristics, utilities and biases of studies related to malignancies using Google Trends: a systematic review
**ABSTRACT**			
Abstract	2	See the PRISMA 2020 for Abstracts checklist.	Background<br>Google Trends (GT) is a freely-available tool presenting Google search statistics. GT is becoming a feasible tool to analyze the interest of Google users in different health phenomena. However, there are a few reviews on GT, and none has contributed specifically to oncology. We aimed to systematically characterize studies related to oncology using GT to describe its utilities and biases.<br>Methods<br>We included all studies utilizing GT to analyze Google searches related to malignancies. We excluded studies written in non-English. The search was done on the PubMed engine on 1st August 2022. We used the following search input: "Google trends" AND ("oncology" OR "cancer" OR "malignancy" OR "tumor" OR "lymphoma" OR "multiple myeloma" OR "leukemia"). We analyzed the following sources of bias: 1) using search terms instead of topics, 2) lack of confrontation with real-world data, and 3) absence of a sensitivity analysis. We performed descriptive statistics.<br>Results<br>A total of n=85 articles were included. We classified n=19 (22.4%) studies as related to prophylaxis, n=17 (20.0%) as an awareness event, n=9 (10.6%) as celebrity-related, n=11 (12.9%) related to COVID-19 and n=40 (47.1%) as others. The most frequently analyzed cancers were: breast (n=28), prostate (n=26), lung, and colorectal (both: n=18). n=79 (92.9%) of the studies provided all search input details to reproduce their results, and n=34 (40%.0) studies confronted GT statistics with real-world data. Authors of only n = 9 (10.6%) studies performed sensitivity analysis.<br>Conclusions<br>The studies in this systematic review varied regarding topics, search strategy, and statistical methods. Most researchers provided reproducible search inputs, but many studies lacked sensitivity analysis.Scientists using GT for medical research should ensure the quality of studies by 1) providing a search strategy to reproduce results, 2) preferring using topics instead of search terms, and 3) performing robust statistical calculations and sensitivity analysis.
**INTRODUCTION**			
Rationale	3	Describe the rationale for the review in the context of existing knowledge.	To date, a few reviews characterized medical research utilizing GT. None of them has explicitly contributed to oncology. The systematic review may reveal the utility of GT in cancer-related studies, its limitations, and good practices for future studies.

Supplementary File 1

PRISMA 2020 Checklist

Section and Topic	Item #	Checklist item	Location where item is reported
Objectives	4	Provide an explicit statement of the objective(s) or question(s) the review addresses.	We aimed to systematically characterize studies related to oncology using GT to describe its utilities and biases.
**METHODS**			
Eligibility criteria	5	Specify the inclusion and exclusion criteria for the review and how studies were grouped for the syntheses.	Here, we included all studies utilizing GT to analyze queries related to malignancies, either solid mass tumors or blood cancers. We excluded studies utilizing only other Google tools e.g. Google AdWords, and those written in non-English.
Information sources	6	Specify all databases, registers, websites, organisations, reference lists and other sources searched or consulted to identify studies. Specify the date when each source was last searched or consulted.	We used the PubMed search engine to search for studies about oncology-related GT analysis. The search was done on the PubMed engine on 1st August 2022.
Search strategy	7	Present the full search strategies for all databases, registers and websites, including any filters and limits used.	The PubMed search input was the following: "Google trends" AND ("oncology" OR "cancer" OR "malignancy" OR "tumor" OR "lymphoma" OR "multiple myeloma" OR "leukemia").
Selection process	8	Specify the methods used to decide whether a study met the inclusion criteria of the review, including how many reviewers screened each record and each report retrieved, whether they worked independently, and if applicable, details of automation tools used in the process.	Two authors (J.C. and M.K.) independently screened titles and abstracts of potential articles. All discrepancies were refereed by PS. The PRISMA flow diagram is presented in Figure 1. We obtained n = 120 pieces for a title and abstract screening. Further, we excluded n = 33 records, n = 2 were additionally excluded because of inaccessible full-version text, and n = 85 articles were thoroughly read. Finally, we included n = 85 for the final review.
Data collection process	9	Specify the methods used to collect data from reports, including how many reviewers collected data from each report, whether they worked independently, any processes for obtaining or confirming data from study investigators, and if applicable, details of automation tools used in the process.	The included articles were carefully read and analyzed by one of the authors (J.C.), who retrieved all essential details. We collected the following details: title, doi code, authors' countries, year of publication, aim(s), aims classification, study category (prophylaxis, awareness month, celebrity death, COVID-19, others), malignancies considered, analysis of topics or search terms, regions and period analyzed, statistical reproducibility method used, confrontation with real-world data, principal findings, sensitivity analysis.
Data items	10a	List and define all outcomes for which data were sought. Specify whether all results that were compatible with each outcome domain in each study were sought (e.g. for all measures, time points, analyses), and if not, the methods used to decide which results to collect.	We classified aims (causal interference, description, surveillance, other) based on the classification used in the review of Nuti et al. After the initial screening of the included articles, we divided all studies into five specific categories: related to the prophylaxis method, related to an awareness event (e.g. Pink October), related to a celebrity (e.g, death or announcement of suffering from cancer), related to the COVID-19 pandemic and others. Reproducibility of the study was defined as the provision of the authors' search input details (search term/topics used, region analyzed, period analyzed, information about including countries with low or no-low search volume if the region was set to "Worldwide") necessary to reproduce the outcomes.
	10b	List and define all other variables for which data were sought (e.g. participant and intervention characteristics, funding sources). Describe any assumptions made about any missing or unclear information.	The complete characteristics of the studies are available in Supplementary File 2.

Supplementary File 1

PRISMA 2020 Checklist

Section and Topic	Item #	Checklist item	Location where item is reported
Study risk of bias assessment	11	Specify the methods used to assess risk of bias in the included studies, including details of the tool(s) used, how many reviewers assessed each study and whether they worked independently, and if applicable, details of automation tools used in the process.	Two authors (J.C. and M.K.) independently screened titles and abstracts of potential articles. All discrepancies were refereed by PS. (…) Reproducibility of the study was defined as the provision of the authors' search input details (search term/topics used, region analyzed, period analyzed, information about including countries with low or no-low search volume if the region was set to "Worldwide") necessary to reproduce the outcomes.
Effect measures	12	Specify for each outcome the effect measure(s) (e.g. risk ratio, mean difference) used in the synthesis or presentation of results.	We performed descriptive statistics. All categorical variables were reported as numbers (percentage), while all numerical as median (interquartile range).
Synthesis methods	13a	Describe the processes used to decide which studies were eligible for each synthesis (e.g. tabulating the study intervention characteristics and comparing against the planned groups for each synthesis (item #5)).	The PubMed search input was the following: "Google trends" AND ("oncology" OR "cancer" OR "malignancy" OR "tumor" OR "lymphoma" OR "multiple myeloma" OR "leukemia"). Two authors (J.C. and M.K.) independently screened titles and abstracts of potential articles. All discrepancies were refereed by PS. (…) We obtained n = 120 pieces for a title and abstract screening. Further, we excluded n = 33 records, n = 2 were additionally excluded because of inaccessible full-version text, and n = 85 articles were thoroughly read. Finally, we included n = 85 for the final review.
	13b	Describe any methods required to prepare the data for presentation or synthesis, such as handling of missing summary statistics, or data conversions.	We performed descriptive statistics. All categorical variables were reported as numbers (percentage), while all numerical as median (interquartile range). Data manipulation, calculations and visualizations were performed using R-programming language 3.6.1 (R Foundation, Vienna, Austria)
	13c	Describe any methods used to tabulate or visually display results of individual studies and syntheses.	Data manipulation, calculations and visualizations were performed using R-programming language 3.6.1 (R Foundation, Vienna, Austria)
	13d	Describe any methods used to synthesize results and provide a rationale for the choice(s). If meta-analysis was performed, describe the model(s), method(s) to identify the presence and extent of statistical heterogeneity, and software package(s) used.	We performed descriptive statistics. All categorical variables were reported as numbers (percentage), while all numerical as median (interquartile range).
	13e	Describe any methods used to explore possible causes of heterogeneity among study results (e.g. subgroup analysis, meta-regression).	Studies utilizing GT are retrospective and involve aggregated data of Google users without details, e.g. sex and age. Nuti et al. address two sources of potential bias in GT studies: search strategy and validation of the studies. Here, we analyzed the following sources of bias: 1) using search terms instead of topics, 2) lack of confrontation with real-world data, and 3) absence of a sensitivity analysis.<br>We performed descriptive statistics. All categorical variables were reported as numbers (percentage), while all numerical as median (interquartile range). Data manipulation, calculations and visualizations were performed using R-programming language 3.6.1 (R Foundation, Vienna, Austria)
	13f	Describe any sensitivity analyses conducted to assess robustness of the synthesized results.	Here, we analyzed the following sources of bias: 1) using search terms instead of topics, 2) lack of confrontation with real-world data, and 3) absence of a sensitivity analysis.
Reporting bias assessment	14	Describe any methods used to assess risk of bias due to missing results in a synthesis (arising from reporting biases).	Here, we analyzed the following sources of bias: 1) using search terms instead of topics, 2) lack of confrontation with real-world data, and 3) absence of a sensitivity

Supplementary File 1

PRISMA 2020 Checklist

Section and Topic	Item #	Checklist item	Location where item is reported
			analysis.
Certainty assessment	15	Describe any methods used to assess certainty (or confidence) in the body of evidence for an outcome.	Studies utilizing GT are retrospective and involve aggregated data of Google users without details, e.g. sex and age. Nuti et al. address two sources of potential bias in GT studies: search strategy and validation of the studies. Here, we analyzed the following sources of bias: 1) using search terms instead of topics, 2) lack of confrontation with real-world data, and 3) absence of a sensitivity analysis.<br>We performed descriptive statistics. All categorical variables were reported as numbers (percentage), while all numerical as median (interquartile range). Data manipulation, calculations and visualizations were performed using R-programming language 3.6.1 (R Foundation, Vienna, Austria)
**RESULTS**			
Study selection	16a	Describe the results of the search and selection process, from the number of records identified in the search to the number of studies included in the review, ideally using a flow diagram.	The PRISMA flow diagram is presented in Figure 1. We obtained n = 120 pieces for a title and abstract screening. Further, we excluded n = 33 records, n = 2 were additionally excluded because of inaccessible full-version text, and n = 85 articles were thoroughly read. Finally, we included n = 85 for the final review. (…)We included 85 articles for our final analysis.
	16b	Cite studies that might appear to meet the inclusion criteria, but which were excluded, and explain why they were excluded.	We obtained n = 120 pieces for a title and abstract screening. Further, we excluded n = 33 records, n = 2 were additionally excluded because of inaccessible full-version text, and n = 85 articles were thoroughly read. Finally, we included n = 85 for the final review. (…)We included 85 articles for our final analysis. Ten examples of included research are presented in Table 1.
Study characteristics	17	Cite each included study and present its characteristics.	The complete characteristics of the studies are available in Supplementary File 2. (…)The first cancer-related study utilizing GT was published in 2013, and in the following years, the yearly number of publications rapidly increased and reached n = 20 in both 2020 and 2021 (Figure 2). A total of n = 23 (27.1%) researches were published in a journal dedicated to oncology, n = 9 (10.6%) in the journal about public health issues, n = 8 (9.4%) in dermatological journals, n = 18 (21.2%) in journal with broad scope (e.g., PloS One, BMJ Open, Cureus), and n = 28 (32.9%) in others types of journals. N = 32 (37.6%) articles were published in open-access journals. Over half of the studies had descriptive characters, mainly characterizing temporal and regional trends (Figure 3). E.g., Zhang et al. described the seasonal interest of Google users in "tobacco" and "lung cancer" in Australia, New Zealand, the United Kingdom, and the United State. Approximately 30% of studies had surveillance characteristics. An example of a surveillance study is Brazilian research assessing Google searches related to breast cancer and mammogram and their association with the number of cases and performed mammographies in Brazilian states. Finally, one of six papers assessed causal interference between specific events and Google searches. For instance, Noar et al. analyzed the effects of public figure announcements of diagnosis and/or death due to pancreatic cancer on Google queries (Table 1).<br>We classified n = 19 (22.4%) studies as related to prophylaxis, n = 17 (20.0%) as

Supplementary File 1

PRISMA 2020 Checklist

Section and Topic	Item #	Checklist item	Location where item is reported
			awareness event, n = 9 (10.6%) as celebrity-related, n = 11 (12.9%) related to COVID-19 and n = 40 (47.1%) as others (Figure 4). An example of studies on prophylaxis is the study of Kamiński et al., who found an association between the burden of colorectal cancer and interest in colonoscopy of Google users in n = 60 countries. Several studies analyzed the association between GT statistics and awareness month, e.g., Pink October represents breast cancer awareness month, and March is an awareness month of colorectal cancer in the United States. Announcements of the death of celebrities due to cancer may increase the number of Google queries on cancer, which was reported after the death of Chadwick Boseman due to colorectal cancer (Table 1). In recent years, many researchers analyzed the effects of the COVID-19 pandemic on the interest of Google users in many malignancies. E.g., Adelhoefer et al. found that interest in many malignancies decreased during the pandemic's first months.<br>The most frequently analyzed cancers were: breast cancer (n = 28), prostate cancer (n = 26), lung cancer (n = 18), colorectal cancers (n = 18), skin cancers (n = 16), and cervical cancer (n = 14) (Figure 5).
Risk of bias in studies	18	Present assessments of risk of bias for each included study.	The complete characteristics of the studies are available in Supplementary File 2. (…) We found that n = 17 (20.0%) of studies admitted using GT topics instead of search terms. Overall, n = 24 (28.2%) researches involved GT statistics for all available countries, n = 33 (38.8%) only for the United States, and n = 30 (35.3%) for other countries or combinations of different countries (including combination with the United States). A total of n = 40 (47.1%) studies utilized the most prolonged period available in GT since 1st January 2004.<br>We identified results that most [n = 79 (92.9%)] of the studies provided all search input details to reproduce their results. Overall, n = 34 (40%.0) studies confronted GT statistics with real-world data. Almost two third of studies performed correlation analysis [n = 55 (64.7%)] and over half peaks analysis [n = 43 (50.6%)]. The less popular statistical approach included secular trends analysis [n = 9 (10.6%)], seasonal trends analysis [n = 6 (7.1%)], forecasting [n = 4 (4.7%)], and others [n = 24 (28.2%)].<br>Authors of only n = 9 (10.6%) studies performed sensitivity analysis. Most studies compared RSV of analyzed terms to the search terms both medical and non-medical. Another convincing sensitivity analysis involved comparing results between different countries or between Northern and Southern Hemisphere countries.
Results of individual studies	19	For all outcomes, present, for each study: (a) summary statistics for each group (where appropriate) and (b) an effect estimate and its precision (e.g. confidence/credible interval), ideally using structured tables or plots.	The complete characteristics of the studies are available in Supplementary File 2. (…)We included 85 articles for our final analysis. Ten examples of included research are presented in Table 1. In n = 47 (55.3%) articles, at least one author came from the United States. Further, at least one author in n = 5 articles came from Australia, Brazil, and the Philippines; n = 4 came from Germany, Ireland, Turkey, and the United Kingdom, n = 3 came from China, Italy, Japan, Austria, n = 2 came from Canada, France, India, Malaysia, a n = 1 from Iran, Kuwait, Netherlands, New

Supplementary File 1

PRISMA 2020 Checklist

Section and Topic	Item #	Checklist item	Location where item is reported
			Zealand, Poland, Portugal, Romania, and Spain. No author came from Africa or Central America.<br>The first cancer-related study utilizing GT was published in 2013, and in the following years, the yearly number of publications rapidly increased and reached n = 20 in both 2020 and 2021 (Figure 2). A total of n = 23 (27.1%) researches were published in a journal dedicated to oncology, n = 9 (10.6%) in the journal about public health issues, n = 8 (9.4%) in dermatological journals, n = 18 (21.2%) in journal with broad scope (e.g., PloS One, BMJ Open, Cureus), and n = 28 (32.9%) in others types of journals. N = 32 (37.6%) articles were published in open-access journals. Over half of the studies had descriptive characters, mainly characterizing temporal and regional trends (Figure 3). E.g., Zhang et al. described the seasonal interest of Google users in "tobacco" and "lung cancer" in Australia, New Zealand, the United Kingdom, and the United States. Approximately 30% of studies had surveillance characteristics. An example of a surveillance study is Brazilian research assessing Google searches related to breast cancer and mammogram and their association with the number of cases and performed mammographies in Brazilian states. Finally, one of six papers assessed causal interference between specific events and Google searches. For instance, Noar et al. analyzed the effects of public figure announcements of diagnosis and/or death due to pancreatic cancer on Google queries (Table 1).<br>We classified n = 19 (22.4%) studies as related to prophylaxis, n = 17 (20.0%) as awareness event, n = 9 (10.6%) as celebrity-related, n = 11 (12.9%) related to COVID-19 and n = 40 (47.1%) as others (Figure 4). An example of studies on prophylaxis is the study of Kamiński et al., who found an association between the burden of colorectal cancer and interest in colonoscopy of Google users in n = 60 countries. Several studies analyzed the association between GT statistics and awareness month, e.g., Pink October represents breast cancer awareness month, and March is an awareness month of colorectal cancer in the United States. Announcements of the death of celebrities due to cancer may increase the number of Google queries on cancer, which was reported after the death of Chadwick Boseman due to colorectal cancer (Table 1). In recent years, many researchers analyzed the effects of the COVID-19 pandemic on the interest of Google users in many malignancies. E.g., Adelhoefer et al. found that interest in many malignancies decreased during the pandemic's first months.<br>The most frequently analyzed cancers were: breast cancer (n = 28), prostate cancer (n = 26), lung cancer (n = 18), colorectal cancers (n = 18), skin cancers (n = 16), and cervical cancer (n = 14) (Figure 5).<br>We found that n = 17 (20.0%) of studies admitted using GT topics instead of search terms. Overall, n = 24 (28.2%) researches involved GT statistics for all available countries, n = 33 (38.8%) only for the United States, and n = 30 (35.3%) for other countries or combinations of different countries (including combination with the United States). A total of n = 40 (47.1%) studies utilized the most prolonged period

Supplementary File 1

PRISMA 2020 Checklist

Section and Topic	Item #	Checklist item	Location where item is reported
			available in GT since 1st January 2004.<br>We identified results that most [n = 79 (92.9%)] of the studies provided all search input details to reproduce their results. Overall, n = 34 (40%.0) studies confronted GT statistics with real-world data. Almost two third of studies performed correlation analysis [n = 55 (64.7%)] and over half peaks analysis [n = 43 (50.6%)]. The less popular statistical approach included secular trends analysis [n = 9 (10.6%)], seasonal trends analysis [n = 6 (7.1%)], forecasting [n = 4 (4.7%)], and others [n = 24 (28.2%)].<br>Authors of only n = 9 (10.6%) studies performed sensitivity analysis. Most studies compared RSV of analyzed terms to the search terms both medical and non-medical. Another convincing sensitivity analysis involved comparing results between different countries or between Northern and Southern Hemisphere countries.
Results of syntheses	20a	For each synthesis, briefly summarise the characteristics and risk of bias among contributing studies.	The complete characteristics of the studies are available in Supplementary File 2. (…)We included 85 articles for our final analysis. Ten examples of included research are presented in Table 1. In n = 47 (55.3%) articles, at least one author came from the United States. Further, at least one author in n = 5 articles came from Australia, Brazil, and the Philippines; n = 4 came from Germany, Ireland, Turkey, and the United Kingdom, n = 3 came from China, Italy, Japan, Austria, n = 2 came from Canada, France, India, Malaysia, a n = 1 from Iran, Kuwait, Netherlands, New Zealand, Poland, Portugal, Romania, and Spain. No author came from Africa or Central America.<br>The first cancer-related study utilizing GT was published in 2013, and in the following years, the yearly number of publications rapidly increased and reached n = 20 in both 2020 and 2021 (Figure 2). A total of n = 23 (27.1%) researches were published in a journal dedicated to oncology, n = 9 (10.6%) in the journal about public health issues, n = 8 (9.4%) in dermatological journals, n = 18 (21.2%) in journal with broad scope (e.g., PloS One, BMJ Open, Cureus), and n = 28 (32.9%) in others types of journals. N = 32 (37.6%) articles were published in open-access journals. Over half of the studies had descriptive characters, mainly characterizing temporal and regional trends (Figure 3). E.g., Zhang et al. described the seasonal interest of Google users in "tobacco" and "lung cancer" in Australia, New Zealand, the United Kingdom, and the United States. Approximately 30% of studies had surveillance characteristics. An example of a surveillance study is Brazilian research assessing Google searches related to breast cancer and mammogram and their association with the number of cases and performed mammographies in Brazilian states. Finally, one of six papers assessed causal interference between specific events and Google searches. For instance, Noar et al. analyzed the effects of public figure announcements of diagnosis and/or death due to pancreatic cancer on Google queries (Table 1).<br>We classified n = 19 (22.4%) studies as related to prophylaxis, n = 17 (20.0%) as awareness event, n = 9 (10.6%) as celebrity-related, n = 11 (12.9%)

Supplementary File 1

PRISMA 2020 Checklist

Section and Topic	Item #	Checklist item	Location where item is reported
			related to COVID-19 and n = 40 (47.1%) as others (Figure 4). An example of studies on prophylaxis is the study of Kamiński et al., who found an association between the burden of colorectal cancer and interest in colonoscopy of Google users in n = 60 countries. Several studies analyzed the association between GT statistics and awareness month, e.g., Pink October represents breast cancer awareness month, and March is an awareness month of colorectal cancer in the United States. Announcements of the death of celebrities due to cancer may increase the number of Google queries on cancer, which was reported after the death of Chadwick Boseman due to colorectal cancer (Table 1). In recent years, many researchers analyzed the effects of the COVID-19 pandemic on the interest of Google users in many malignancies. E.g., Adelhoefer et al. found that interest in many malignancies decreased during the pandemic's first months.<br>The most frequently analyzed cancers were: breast cancer (n = 28), prostate cancer (n = 26), lung cancer (n = 18), colorectal cancers (n = 18), skin cancers (n = 16), and cervical cancer (n = 14) (Figure 5).<br>We found that n = 17 (20.0%) of studies admitted using GT topics instead of search terms. Overall, n = 24 (28.2%) researches involved GT statistics for all available countries, n = 33 (38.8%) only for the United States, and n = 30 (35.3%) for other countries or combinations of different countries (including combination with the United States). A total of n = 40 (47.1%) studies utilized the most prolonged period available in GT since 1st January 2004.<br>We identified results that most [n = 79 (92.9%)] of the studies provided all search input details to reproduce their results. Overall, n = 34 (40%.0) studies confronted GT statistics with real-world data. Almost two third of studies performed correlation analysis [n = 55 (64.7%)] and over half peaks analysis [n = 43 (50.6%)]. The less popular statistical approach included secular trends analysis [n = 9 (10.6%)], seasonal trends analysis [n = 6 (7.1%)], forecasting [n = 4 (4.7%)], and others [n = 24 (28.2%)].<br>Authors of only n = 9 (10.6%) studies performed sensitivity analysis. Most studies compared RSV of analyzed terms to the search terms both medical and non-medical. Another convincing sensitivity analysis involved comparing results between different countries or between Northern and Southern Hemisphere countries.
	20b	Present results of all statistical syntheses conducted. If meta-analysis was done, present for each the summary estimate and its precision (e.g. confidence/credible interval) and measures of statistical heterogeneity. If comparing groups, describe the direction of the effect.	A total of n = 23 (27.1%) researches were published in a journal dedicated to oncology, n = 9 (10.6%) in the journal about public health issues, n = 8 (9.4%) in dermatological journals, n = 18 (21.2%) in journal with broad scope (e.g., PloS One, BMJ Open, Cureus), and n = 28 (32.9%) in others types of journals. N = 32 (37.6%) articles were published in open-access journals. Over half of the studies had descriptive characters, mainly characterizing temporal and regional trends (Figure 3). E.g., Zhang et al. described the seasonal interest of Google users in "tobacco" and "lung cancer" in Australia, New Zealand, the United Kingdom, and the United States. Approximately 30% of studies had surveillance characteristics. An example

Supplementary File 1

PRISMA 2020 Checklist

Section and Topic	Item #	Checklist item	Location where item is reported
			of a surveillance study is Brazilian research assessing Google searches related to breast cancer and mammogram and their association with the number of cases and performed mammographies in Brazilian states. Finally, one of six papers assessed causal interference between specific events and Google searches. For instance, Noar et al. analyzed the effects of public figure announcements of diagnosis and/or death due to pancreatic cancer on Google queries (Table 1).<br>We classified n = 19 (22.4%) studies as related to prophylaxis, n = 17 (20.0%) as awareness event, n = 9 (10.6%) as celebrity-related, n = 11 (12.9%) related to COVID-19 and n = 40 (47.1%) as others (Figure 4). An example of studies on prophylaxis is the study of Kamiński et al., who found an association between the burden of colorectal cancer and interest in colonoscopy of Google users in n = 60 countries. Several studies analyzed the association between GT statistics and awareness month, e.g., Pink October represents breast cancer awareness month, and March is an awareness month of colorectal cancer in the United States. Announcements of the death of celebrities due to cancer may increase the number of Google queries on cancer, which was reported after the death of Chadwick Boseman due to colorectal cancer (Table 1). In recent years, many researchers analyzed the effects of the COVID-19 pandemic on the interest of Google users in many malignancies. E.g., Adelhoefer et al. found that interest in many malignancies decreased during the pandemic's first months.<br>The most frequently analyzed cancers were: breast cancer (n = 28), prostate cancer (n = 26), lung cancer (n = 18), colorectal cancers (n = 18), skin cancers (n = 16), and cervical cancer (n = 14) (Figure 5).<br>We found that n = 17 (20.0%) of studies admitted using GT topics instead of search terms. Overall, n = 24 (28.2%) researches involved GT statistics for all available countries, n = 33 (38.8%) only for the United States, and n = 30 (35.3%) for other countries or combinations of different countries (including combination with the United States). A total of n = 40 (47.1%) studies utilized the most prolonged period available in GT since 1st January 2004.<br>We identified results that most [n = 79 (92.9%)] of the studies provided all search input details to reproduce their results. Overall, n = 34 (40%.0) studies confronted GT statistics with real-world data. Almost two third of studies performed correlation analysis [n = 55 (64.7%)] and over half peaks analysis [n = 43 (50.6%)]. The less popular statistical approach included secular trends analysis [n = 9 (10.6%)], seasonal trends analysis [n = 6 (7.1%)], forecasting [n = 4 (4.7%)], and others [n = 24 (28.2%)].<br>Authors of only n = 9 (10.6%) studies performed sensitivity analysis. Most studies compared RSV of analyzed terms to the search terms both medical and non-medical. Another convincing sensitivity analysis involved comparing results between different countries or between Northern and Southern Hemisphere countries.

Supplementary File 1

PRISMA 2020 Checklist

Section and Topic	Item #	Checklist item	Location where item is reported
	20c	Present results of all investigations of possible causes of heterogeneity among study results.	We found that n = 17 (20.0%) of studies admitted using GT topics instead of search terms. Overall, n = 24 (28.2%) researches involved GT statistics for all available countries, n = 33 (38.8%) only for the United States, and n = 30 (35.3%) for other countries or combinations of different countries (including combination with the United States). A total of n = 40 (47.1%) studies utilized the most prolonged period available in GT since 1st January 2004.<br>We identified results that most [n = 79 (92.9%)] of the studies provided all search input details to reproduce their results. Overall, n = 34 (40%.0) studies confronted GT statistics with real-world data. Almost two third of studies performed correlation analysis [n = 55 (64.7%)] and over half peaks analysis [n = 43 (50.6%)]. The less popular statistical approach included secular trends analysis [n = 9 (10.6%)], seasonal trends analysis [n = 6 (7.1%)], forecasting [n = 4 (4.7%)], and others [n = 24 (28.2%)].<br>Authors of only n = 9 (10.6%) studies performed sensitivity analysis. Most studies compared RSV of analyzed terms to the search terms both medical and non-medical. Another convincing sensitivity analysis involved comparing results between different countries or between Northern and Southern Hemisphere countries.
	20d	Present results of all sensitivity analyses conducted to assess the robustness of the synthesized results.	We identified results that most [n = 79 (92.9%)] of the studies provided all search input details to reproduce their results. Overall, n = 34 (40%.0) studies confronted GT statistics with real-world data. Almost two third of studies performed correlation analysis [n = 55 (64.7%)] and over half peaks analysis [n = 43 (50.6%)]. The less popular statistical approach included secular trends analysis [n = 9 (10.6%)], seasonal trends analysis [n = 6 (7.1%)], forecasting [n = 4 (4.7%)], and others [n = 24 (28.2%)].<br>Authors of only n = 9 (10.6%) studies performed sensitivity analysis. Most studies compared RSV of analyzed terms to the search terms both medical and non-medical. Another convincing sensitivity analysis involved comparing results between different countries or between Northern and Southern Hemisphere countries.
Reporting biases	21	Present assessments of risk of bias due to missing results (arising from reporting biases) for each synthesis assessed.	Not applicable.
Certainty of evidence	22	Present assessments of certainty (or confidence) in the body of evidence for each outcome assessed.	The complete characteristics of the studies are available in Supplementary File 2. (…) We identified results that most [n = 79 (92.9%)] of the studies provided all search input details to reproduce their results. Overall, n = 34 (40%.0) studies confronted GT statistics with real-world data. Almost two third of studies performed correlation analysis [n = 55 (64.7%)] and over half peaks analysis [n = 43 (50.6%)]. The less popular statistical approach included secular trends analysis [n = 9 (10.6%)], seasonal trends analysis [n = 6 (7.1%)], forecasting [n = 4 (4.7%)], and others [n = 24 (28.2%)].<br>Authors of only n = 9 (10.6%) studies performed sensitivity analysis. Most studies

Supplementary File 1

PRISMA 2020 Checklist

Section and Topic	Item #	Checklist item	Location where item is reported
			compared RSV of analyzed terms to the search terms both medical and non-medical. Another convincing sensitivity analysis involved comparing results between different countries or between Northern and Southern Hemisphere countries.
**DISCUSSION**			
Discussion	23a	Provide a general interpretation of the results in the context of other evidence.	Our study found that the number of studies utilizing GT in oncology has constantly been increasing. Most of the included papers come from the Western World, and we did not find any papers from Africa or Central America. Moreover, many of the available studies successfully use data from the Global South, e.g., Africa. Previous studies observed that many developing countries have a low search volume for many popular health topics, such as pain or diets. It could be related to the lower access to the Internet in the Global South.<br>Furthermore, developing countries have far less productive research facilities than the Western World. However, the number of people using the Internet is increasing the fastest in developing countries. Therefore, we may expect more data about Google searches in these regions to be available shortly.<br>The included articles were published in journals with various scopes. Moreover, approximately one of three papers were published in an open-access journal. It is tempting to hypothesize that articles using GT are usually held as a curiosity and often published in wide-scope journals.<br>In our systematic review, we utilized the classification of GT articles used previously by Nuti et al. Here, we observed that most of the oncological studies utilizing GT had descriptive character, and a total of less than 20% analyzed causal interference and up to 30% surveillance. Nuti et al. reported that 39% of studies used GT for description, 34% for surveillance, and 27% for causal interference. The proportion between both reviews seems similar. However, the review by Nuti et al. included all GT studies on health phenomena from 2009 to 2013, while we had narrower inclusion criteria but included all studies until mid-2022. Nuti et al. defined descriptive studies as "aimed to describe temporal or geographic trends and general relationships, without reference to a hypothesized causal relationship". For this reason, we assume that descriptive studies are generally easier to perform by researchers, which explains their popularity.<br>The analyzed studies vary in terms of study category, which presents many ideas for utilizing GT in oncology-related studies.<br>A large group of papers took the form of prophylaxis studies. They mainly focused on assessing the interest in oncological screenings and a wide range of preventive activities and risk factors of the common neoplasms. Most studies analyzed the interest of Google users in cancer screenings in the USA. These studies concerned, among others, topics related to the prevention of melanoma and various risk factors of skin cancer known from the literature. (…)<br>An interesting direction in using GT was studying the relationship between the

Supplementary File 1

PRISMA 2020 Checklist

Section and Topic	Item #	Checklist item	Location where item is reported
			getting cancer from a celebrity and the global interest in this particular malignancy. In general, it is observed that the diagnosis, and especially the premature death of a celebrity combined with the announcement of this fact to the media, significantly increases the interest in a given cancer. Examples of such relationships were the increased interest in colorectal cancer and colon cancer screening associated with the death of Chadwick Boseman (actor), the diagnosis and death of Patrick Swayze (actor) and Steve Jobs (Apple co-founder), due to pancreatic cancer or the diagnosis of the prostate cancer in Ben Stiller (actor). In addition, the analysis of the effect of the cancer-related death of a celebrity is not the only example in the scientific literature. Another example is the death of Harold Allen Ramisz (actor) due to complications of an autoimmune inflammatory vasculitis.<br>Another direction was to use GT to examine whether social campaigns or cancer awareness month are associated with the interest fluctuations of Google users in specific malignancies. Firstly, GT is a widely available, free tool to check the importance of health promotion events. Several papers' data showed a correlation between the cancer awareness campaign and the increased public interest in oncological screening. Such observations have been reported for Pink October and breast screening in Malaysia. Furthermore, several studies have confirmed that events such as Breast Cancer Awareness Month were associated with increased interest in this topic among Google users. Studies confirmed that, compared to other months, there was an increase in searches for topics related to breast cancer and mammography in October in all countries.<br>However, similar campaigns for lung or prostate cancer did not show a similar relationship. Patel et al. observed that cancer awareness campaigns aimed at men are not associated with a significant increase in interest in topics related to screening, risk factors, or cancer itself. Gender may be the potential cause of these observations. Women, as Internet users, are usually described as more likely to search for health topics and to be more aware of Internet use in this area. Interestingly, studies analyzing data from all countries show the lack of intended effectiveness and increased interest in many cancers.On the contrary, studies analyzing national data, such as from Brazil and New Zealand, described an increased interest in such cancers as glaucoma, prostate, and lung. Finally, not only awareness events related to malignancies have been investigated with GT, but also a similar paper on the effect of World Sepsis Day was conducted.<br>Several studies analyzed the interest of Google users in cancers during the COVID-19 pandemic and compared the pre-pandemic period with the first months or years of the pandemic. The analyzed studies showed a decrease in interest in many cancers and their screening programs during the advent of a novel virus. The decreases in analyzed search terms' RSVs were generally consistent with the simultaneous decrease in the number of diagnostic procedures or new cancer diagnoses. These studies were feasible examples of using GT in emerging public health problems. GT was previously used to analyze outbreaks of infectious

Supplementary File 1

PRISMA 2020 Checklist

Section and Topic	Item #	Checklist item	Location where item is reported
			diseases. Here, we presented a series of GT studies showing how COVID-19 could have diminished the importance of cancer awareness, which may lead to increased excess mortality during the pandemic. Notably, some GT observations were proven by real-world data.<br>Interestingly, the most commonly analyzed cancers are those 1) with screening programs or 2) with the highest prevalence worldwide. The search terms representing rare malignancies could be written by a small population of Google users. For this reason, the trends of rarely typed search terms could be susceptible to irregular fluctuations without specific secular or seasonal patterns. The trends of search terms with low search volume could be problematic in statistical analysis and interpretation of the results. That problem could be prevailed by matching the search terms of a topic in GT search engine to include more significant regions and all queries related to the topic. However, only one in five included GT studies admitted to using topics instead of search terms.<br>Correlation and peaks analyses were the most prevalent among included studies. These methods are widely used and are easy to understand by readers. However, correlation analysis allows only to detection of associations, and many outcomes may represent incidental findings if not confronted with real-world data or strengthened by sensitivity analysis. Regrettably, a few studies analyze secular or seasonal patterns, and only four performed forecasting. Our findings are similar to the results of Mavragani et al., who also found that correlation analysis was the most prevalent and forecasting analysis the least prevalent among studies utilizing GT for analyzing health phenomena.<br>Forty percent of papers confronted their results with real-world, and only nine reviewed studies applied sensitivity analysis. In our opinion, many GT pieces of research, without being compared with real-world data (e.g. epidemiological data) or without appropriate sensitivity analyses, are only a form of curiosity. The GT data cannot be fundamental to new recommendations or epidemiological/e-health tools. In that form, the data from GT provide only a curiosity, giving, at most, a new perspective on some epidemiological issues. However, GT may provide insight into health phenomena that would require large-scale observations with uncertain clinical significance.
	23b	Discuss any limitations of the evidence included in the review.	Our systematic review concerned retrospective studies on various topics, making it difficult to classify them uniformly. (…)In our review, we did not analyze whether the exact results of the papers could be reproduced or whether the authors drew the appropriate conclusions.
	23c	Discuss any limitations of the review processes used.	For this reason, we performed only descriptive statistics. Furthermore, there are no standards for conducting systematic reviews of studies utilizing GT data. The novelty of the tool makes the researcher rely on their diligence. However, this is one of the first systematic reviews of GT ever, and the methodological practice is just being formed.

Supplementary File 1

PRISMA 2020 Checklist

Section and Topic	Item #	Checklist item	Location where item is reported
	23d	Discuss implications of the results for practice, policy, and future research.	To the best of our knowledge, the paper is the first to explore GT's utility, specifically in oncology and hematology. We characterized various studies presenting the readers' feasibility of GT in research related to oncology and hematology. We pointed methodological weaknesses of the analyzed reports. Papers using GT data should not only report search inputs appropriately and report them as proposed by Nuti et al.. Furthermore, using topics allow to inclusion of more regions, which increases the value of the results. We also suggest that the GT data should be either confronted with real-world data or, if seasonality is analyzed, searches in both hemispheres should be compared. Another interesting approach to sensitivity analysis in GT studies was presented by Gillis et al., who proposed benchmark search terms to exclude random seasonal patterns related to the academic year. Finally, it is worth mentioning that there are no strict rules for sensitivity analysis, but even re-analysis, with excluding certain period, region, etc., increase confidence in obtained results.
**OTHER INFORMATION**			
Registration and protocol	24a	Provide registration information for the review, including register name and registration number, or state that the review was not registered.	This is a systematic review; thus, the paper did not require Ethical Committee approval.
	24b	Indicate where the review protocol can be accessed, or state that a protocol was not prepared.	We followed the PRISMA 2020 guidelines on systematic review. The PRISMA 2020 checklist is presented in Supplementary File 1.
	24c	Describe and explain any amendments to information provided at registration or in the protocol.	Not applicable.
Support	25	Describe sources of financial or non-financial support for the review, and the role of the funders or sponsors in the review.	External funding<br>None
Competing interests	26	Declare any competing interests of review authors.	Conflict of interest<br>Nothing to disclosure
Availability of data, code and other materials	27	Report which of the following are publicly available and where they can be found: template data collection forms; data extracted from included studies; data used for all analyses; analytic code; any other materials used in the review.	We followed the PRISMA 2020 guidelines on systematic review. The PRISMA 2020 checklist is presented in Supplementary File 1. (…) We used the PubMed search engine to search for studies about oncology-related GT analysis. The search was done on the PubMed engine on 1st August 2022.

From: Page MJ, McKenzie JE, Bossuyt PM, Boutron I, Hoffmann TC, Mulrow CD, et al. The PRISMA 2020 statement: an updated guideline for reporting systematic reviews. BMJ 2021;372:n71. doi: 10.1136/bmj.n71
